# Supplementary material for: Transgenic Maize Has Insignificant Effects on the Diversity of Arthropods: A 3-Year Study
Source: Plants (Basel). 2022 Aug 30;11(17):2254. doi: 10.3390/plants11172254 (PMC9460771; doi:10.3390/plants11172254)
Supplement: Supplementary file 1 [file plants-11-02254-s001.zip › Supplementary material 1.pdf]

Supplementary Material

1 Supplementary Tables

Table S1. Sampling dates from 2015 to 2017

Plant-inhabiting arthropods			Ground-dwelling arthropods		
2015	2016	2017	2015	2016	2017
6.24	6.16	6.15		7.3	
7.1	6.23	6.22	7.1		6.22
7.9	7.1	6.29			
7.15	7.7	7.7	7.15		
7.21	7.14	7.13		7.14	
7.29	7.2	7.2	7.29		7.7
8.5	7.28	7.27		7.28	
8.12	8.4	8.13	8.12		
8.19	8.1	8.16		8.1	8.17
8.26	8.17	8.24	8.26		
9.2	8.23	8.31		8.24	9.1
9.9	9.1	9.7	9.9		
9.16	9.7	9.15		9.7	9.15
	9.14	9.22	9.23		
	9.22			9.22	

Table S2. Orders, family and parts of identified patterns of the arthropod community in maize field

Orders	Families	Identified Species
Scolopendrida	Scolopendridae	
Scutigeromorpha	Scutigeridae	
Polydesmida	Polydesmidae	
Acarina	Tetranychidae	
	Ixodidae	
Araneae	Lycosidae	*Pirata subpiraticus*
	Agelenidae	
	Linyphiidae	
	Theridiidae	
	Salticidae	
	Thomisidae	*Misumenops tricupiuatus, Synema globosum*
	Araneidae	
	Pholcidae	
Collembola	Entomobryidae	*Entomobrya sauteriBorner*
	Sminthuridae	
Odonata	Libellulidae	
	Coenagriidae	

Ephemeroptera	Ephemeridae	
Mantodea	Mantidae	*Mantis religiosa*
Orthoptera	Acridiidae	*Oxya agavisa, Oedaleus asiaticus, Oedaleus infernalis, Chorthippus aethalinus*
	Tettigidae	
	Tettigoniidae	*Tettigonia chinensis, Gampsocleis sedakovii*
	Gryllidae	*Teleogryllus emma*
	Gryllotalpidae	*Gryllotalpa orientalis*
	Tridactylidae	
Dermaptera	Labiduridae	
Hemiptera	Cercopidae	
	Cicadellidae	*Cicadella viridis*
	Aphididae	*Rhopalosiphum maidis*
	Aleyrodidae	*Trialeurodes vaporariorum*
	Anthocoridae	*Orius sauteri*
	Miridae	*Trigonotylus ruficornis*
	Reduviidae	*Sphedanolestes impressicollis*
	Lygaeidae	
	Pentatomidae	*Eurydema gebleri*
Corrodentia	Psocidae	

Thysanoptera	Thripidae	*Haplothrips aculeatus, Franklinielle tenuicornis*
Coleoptera	Cicindelidae	*Cicindela gemmata*
	Carabidae	*Chlaenius bioculatus, Isiocarabus fiduciaries, Dolichus halensis*
	Staphylinidae	
	Pselaphidae	
	Elateridae	*Pleonomus canaliculatus*
	Cucujidae	
	Nitidulidae	
	Coccinellidae	*Harmonia axyridis, Propylaea japonica*
	Meloidae	
	Tenebrionidae	
	Geotrupidae	
	Melolonthidae	
	Rutelidae	*Anomala corpulenta*
	Chrysomelidae	
	Galerucidae	*Monolepta hieroglyphica*
	Halticidae	
	Curculionoidae	

Neuroptera	Chrysopidae	*Chrysopa pallen, Chrysopa Formosa, Chrysopa yatsumatsui*
Lepidoptera	Gracilariidae	
	Psychidae	
	Cochlidiidae	*Cnidocampa flavescens*
	Pyralidae	*Ostrinia furnacalis*
	Lasiocampidae	
	Geometridae	
	Noctuidae	*Mythimna separata, Helicoverpa armigera, Agrotis ypsilon*
	Lymantriidae	
Diptera	Tipulidae	
	Culicidae	
	Mycetophilidae	
	Cecidomyiidae	
	Tabanidae	
	Asilidae	
	Syrphidae	*Episyrphus balteatus*
	Drosophilidae	
	Muscidae	
	Calliphoridae	

	Larvaevoridae	
	Agromyzidae	
	Trypetidae	
Hymenoptera	Tenthredinidae	
	Siricidae	*Tremex fuscicornis*
	Ichneumonidae	
	Trichogrammatidae	*Trichogramma ostriniae*
	Bethylidae	
	Formicidae	*Camponotus japionicus*
	Eumenidae	
	Tiphiidae	
	Vespidae	
	Polistidae	
	Apidae	*Apis cerana*

Table S3. From 2015 to 2017, the cumulative number of main arthropod groups in the field of cultivated variety XianYu335 (XY), non-transgenic recipient variety DBN318 (NT), transgenic maize DBN9868 (T1) and DBN9936 (T2).

Year	Maize	Aphids	Leaf beetles	Lepidopterans	Ladybirds	Spiders	Lacewings	Parasitic wasps
2015	XY	798±91.3	2310±215	30.7±8.7	196.7±7.1	88.7±9.4	64.7±9.6	7.3±3.5
	NT	862±151.7	2419.3±46.4	21.3±4.4	222.7±33.8	90.7±9.8	58.7±12.7	2.7±2.7
	T1	922±94.3	2422±116.9	21.3±1.8	232±21.9	77.3±6.4	55.3±4.8	5.3±0.7
2015	XY	1031.3±124.3	2058±112.2	30.7±10.9	211.3±14.8	118.7±7.7	50.7±8.2	6.7±0.7
	NT	1526.7±236.6	2684.7±66.6	24±7	215.3±24.1	105.3±4.8	64.7±5.7	6±4.2
	T2	1668±413.1	2462±179.5	2.7±1.8	232±13.3	87.3±9.3	61.3±4.1	6±4.2
2016	XY	7621.3±650.7	1636.7±164.7	12.7±2.4	402±64	155.3±26.3	93.3±20.5	14.7±1.8
	NT	30710.7±549.5	1245.3±42.1	10.7±5.2	475.3±17.9	104.7±8.1	115.3±3.5	13.3±2.7
	T1	31945.3±1525.2	1482.7±155.3	6.7±1.8	620.7±51.2	126.7±10.5	111.3±2.4	14.7±1.8
2017	XY	5444±607.7	40±3.5	26.7±2	221.3±21.9	88±3.5	39±3.5	8±0.6
	NT	5408.3±324.9	51±4.6	24±6	211.3±34	97.7±12.1	41±7	9±3.8
	T2	5357±148.5	42.3±3.8	3.3±1.7	235.7±8.7	87±8.5	51±6.5	7.3±1.8

Table S4. The expression levels of Cry1Ab, EPSPS, and PAT proteins in maize materials determined by ELISA (μg/g·dwt)

Maize material	DBN9868 (T1)		DBN9936 (T2)		DBN318 (NT)		
Protein	PAT	EPSPS	Cry1Ab	EPSPS	PAT	EPSPS	Cry1Ab
roots	12.56±1.30	52.90± 13.75	1.32±0.24	90.92± 15.98	NA	NA	NA
stems	4.50±1.17	51.48± 13.25	1.19±0.12	40.10±5.36	NA	NA	NA
leaves	34.10±6.21	954.34± 46.45	11.75±1.58	921.48± 198.14	NA	NA	NA
seeds	1.24±0.08	12.46±1.39	0.31±0.04	20.07±3.53	NA	NA	NA

Note: NA indicates that the value is lower than the valid detection range of the standard curve, and not detected

2 Supplementary Figures

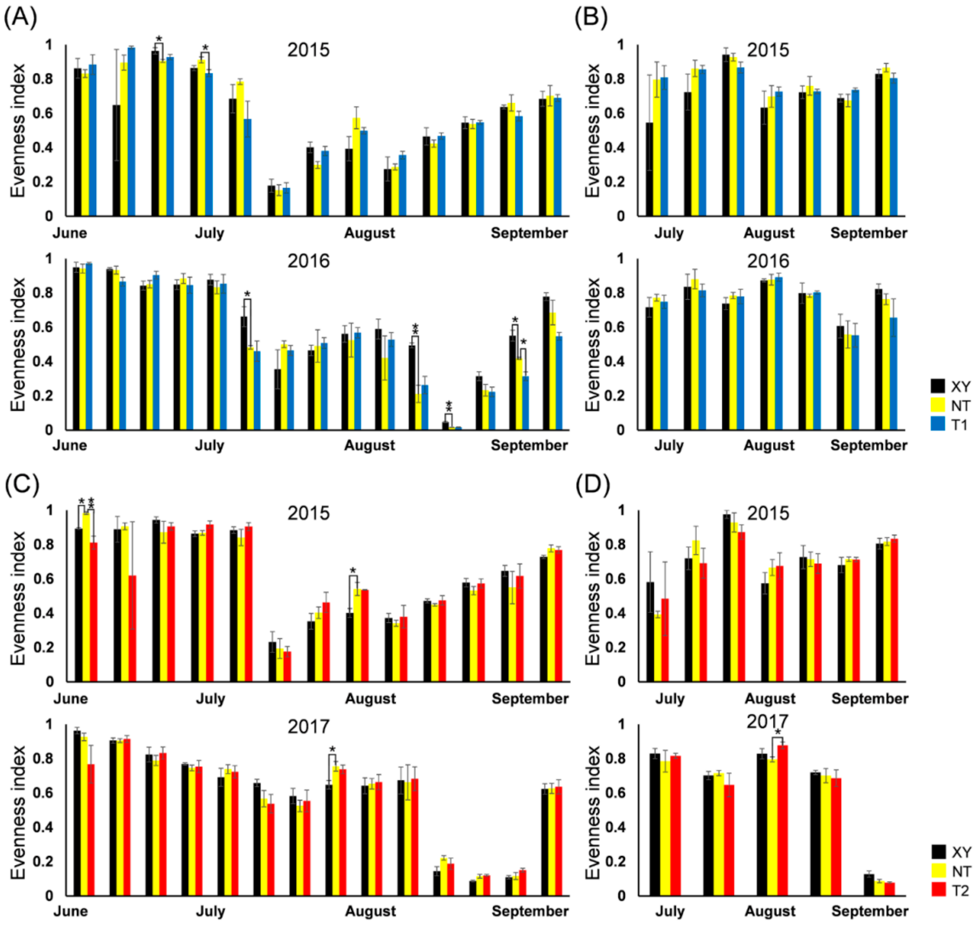

Figure S1. From 2015 to 2017, dynamics of Pielou evenness index (*J*) of arthropod community in the fields of cultivated variety XianYu335 (XY), non-transgenic recipient variety DBN318 (NT), transgenic maize DBN9868 (T1) and DBN9936 (T2). (**A**) The *J* of plant-inhabiting arthropods in XY, NT, and T1; (**B**) the *J* of ground-dwelling arthropods in XY, NT, and T1; (**C**) the *J* of plant-inhabiting arthropods in XY, NT, and T2; (**D**) the *J* of ground-dwelling arthropods in XY, NT, and T2. Y-axis: Mean ± SE (n = 3) of *J* at each sampling point. We only compared XY with NT and NT with T1 or T2 by using a Student's *t*-test. Asterisks denote significant differences, where $p< 0.05$ (*), $p < 0.01$ (**).

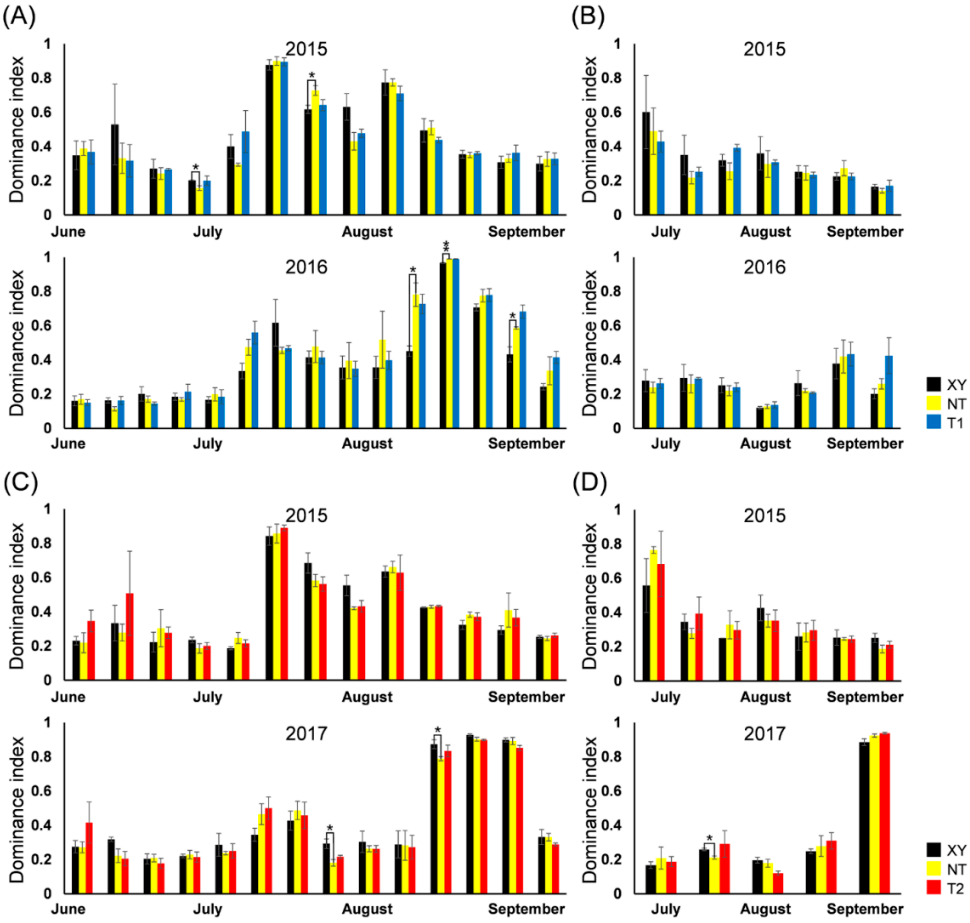

Figure S2. From 2015 to 2017, dynamics of the dominance index (*C*) of arthropod communities in the fields of cultivated variety XianYu335 (XY), non-transgenic recipient variety DBN318 (NT), transgenic maize DBN9868 (T1) and DBN9936 (T2). (**A**) The *C* of plant-inhabiting arthropods in XY, NT, and T1; (**B**) the *C* of ground-dwelling arthropods in XY, NT, and T1; (**C**) the *C* of plant-inhabiting arthropods in XY, NT, and T2; (**D**) the *C* of ground-dwelling arthropods in XY, NT, and T2. Y-axis: Mean ± SE (n = 3) of *C* at each sampling point. We only compared XY with NT and NT with T1 or T2 by using a Student's *t*-test. Asterisks denote significant differences, where $p < 0.05$ (*), $p < 0.01$ (**).

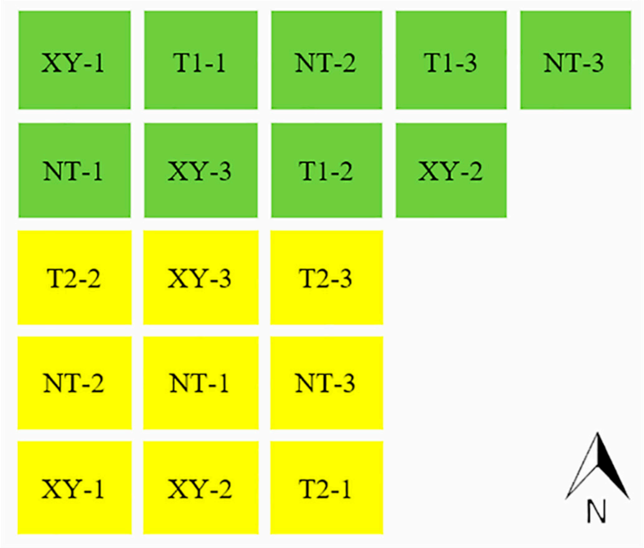

Figure S3. Plots design in 2015. Each variety was replicated three times on 150 m^2 (10 m × 15 m), with corridors of 1.0 m between them. In order to avoid differences caused by heterogeneity of the plot, there were two separate experiments in 2015 with the treatments XY and NT in each of the experiments. The two experimental plots were partially adjacent.

3 Supplementary Materials and Methods

Levels of PAT, EPSPS, and Cry1Ab in maize. The levels of the PAT, EPSPS, and Cry1Ab proteins in roots, stems, leaves, and seeds of maize were quantified using the proprietary ELISA Kit (EnviroLogix Quantiplate Kit, Portland ME, USA), performed according to the manufacturer's instructions. About 500 mg of different fresh tissues of maize at the maturity period were collected from a pool of three randomly selected plants per plot. Before grinding tissues, the samples were washed five times with PBST buffer (made of 137 mM NaCl, 2.7 mM KCl, 10 mM Na_2HPO_4, 2 mM KH_2PO_4, and 0.05% Tween-20, at pH 7.4). Washed tissues were then mixed with 1 mL of extracting buffer (provided with the kits) and ground on ice using an electric grinding rod. The mixture was centrifuged (7000×g, 15 min) and the resulting liquid was used to determine the concentration of PAT, EPSPS, and Cry1Ab protein by assessing the sample's optical densities (ODs), which were measured using the SpectraMax i3x Multi-Mode Detection Platform (Molecular Devices, Sunnyvale, USA). Three experimental replicates were run for each sample type.
